# Effect of Water-Pipe Smoking on the Normal Development of Zebrafish

**DOI:** 10.3390/ijerph182111659

**Published:** 2021-11-06

**Authors:** Zain Zaki Zakaria, Shaima Ahmad Aladwi, Fatiha Benslimane, Enas S. Al-Absi, Mashael Al-Shafai, Huseyin C. Yalcin, Ashraf Khalil, Ala-Eddin Al Moustafa, Maha Al-Asmakh

**Affiliations:** 1 Department of Biomedical Sciences, College of Health Sciences, QU Health, Qatar University, Doha P.O. Box 2713, Qatar; Zain.zakaria@qu.edu.qa (Z.Z.Z.); sa1407179@student.qu.edu.qa (S.A.A.); enas.alabsi@qu.edu.qa (E.S.A.-A.); malshafai@qu.edu.qa (M.A.-S.); 2 Biomedical Research Center, Qatar University, Doha P.O. Box 2713, Qatar; fatiha@qu.edu.qa (F.B.); hyalcin@qu.edu.qa (H.C.Y.); aalmoustafa@qu.edu.qa (A.-E.A.M.); 3 Biomedical and Pharmaceutical Research Unit, QU Health, Qatar University, Doha P.O. Box 2713, Qatar; akhalil@qu.edu.qa; 4 College of Pharmacy, QU Health, Qatar University, Doha P.O. Box 2713, Qatar; 5 College of Medicine, QU Health, Qatar University, Doha P.O. Box 2713, Qatar

**Keywords:** zebrafish, waterpipe smoking, toxicity testing, developmental biology

## Abstract

Background: Among all types of tobacco consumption, Water-Pipe Smoking (WPS) is the most widely used in the Middle East and second-most in several other countries. The effect of WPS on normal development is not yet fully understood, thus the aim of this study is to explore the acute toxicity effects of WPS extract on zebrafish larvae. Methods: In this study, we compared the effects of WPS smoke condensates at concentrations varying from 50 to 200 µg/mL on developmental, cardiac, and behavioural (neurotoxicity) functions. Gene expression patterns of cardiac biomarkers were also evaluated by RT-qPCR. Results: Exposing zebrafish embryos to 50, 100, 150 and 200 µg/mL WPS for three days did not affect the normal morphology of Zebrafish embryos, as the tail flicking, behavioural and locomotion assays did not show any change. However, WPS deregulated cardiac markers including atrial natriuretic peptide (ANP/NPPA) and brain natriuretic peptide (BNP/NPPB). Furthermore, it induced apoptosis in a dose-dependent manner. Conclusion: Our data demonstrate that WPS can significantly affect specific cardiac parameters during the normal development of zebrafish. Further investigations are necessary to elucidate the pathogenic outcome of WPS on different aspects of human life, including pregnancy.

## 1. Introduction

Tobacco smoking is considered a major cause of morbidity and mortality worldwide. According to the World Health Organization it accounts for 6 million deaths each year [1]. Tobacco smoke consists of solid particles and gases. More than 7000 different chemicals have been identified in tobacco smoke and more than 70 of those chemicals are linked to cancer [2]. Among all types of tobacco consumption, including cigarette smoking, waterpipe smoking (WPS), E-cigarettes and smokeless tobacco, WPS is the most widely used in the Middle East and the second-most in many other countries. The highest prevalence of waterpipe smoking is among school/university students across several countries including the United States, especially among Arab Americans, the Arabic Gulf region, Lebanon, and Egypt. In Lebanon, 5–6% of pregnant women reported smoking waterpipe during pregnancy [3,4,5]. WPS is spreading worldwide and is attracting women and youth [4,6]. Many smoking women find it challenging to quit during pregnancy; for example, about 7–9% of pregnant women smoke [5,6]. Since many women continue to smoke WPS and tobacco-related products during pregnancy, the number of fetuses exposed to nicotine continues to grow. While there are very limited studies regarding the effect of WPS on the embryo [7,8,9], our group recently reported that WPS could significantly affect the normal development of chicken embryo at its early stages [10].

On the other hand, it is well known that Zebrafish (Danio rerio) shares 80% of its genes with humans [11] and has been used to predict teratogenicity, toxicity, morphological defects and assess preclinical pharmacological tests [12,13,14]. Around the mid-1900s, zebrafish was introduced as a potential model to study vertebrate development. Since that time, the zebrafish community has developed several techniques involving genetic manipulations and developmental profiling, which aid in understanding many clinical unknowns compounds [12,13]. Currently, zebrafish are widely used as a model for certain human diseases, including cardiovascular [15] and neurodegenerative diseases [16], as well as carcer [17]. Thus, zebrafish may provide insights into the development of smoking-related disorders [18], a cost-effective model for evaluating tobacco smoking and its adverse outcome during the early stage of embryogenesis. Few studies have utilized the zebrafish model to evaluate the effects of smoking exposure [7,19]. Palpant et al. used Zebrafish to examine the cardiac effects of developmental exposure to purified nicotine, conventional cigarette smoke or e-cigarette vapour during the first three days of development [20]. Overall, tobacco and e-cigarette smoke-exposed embryos showed significantly more heart defects compared to the purified nicotine group. Heart defects in embryos exposed to tobacco and e-cigarette smoke were also more severe than in embryos exposed to nicotine alone [20]. However, the effects of WPS on zebrafish development have not been examined. This, combined with the very limited number of studies pertaining to the outcome of WPS on embryogenesis [10], we herein assess the effect of WPS on Zebrafish early stages of development.

All experiments presented in this paper were conducted under the Qatar University’s Institutional Animal Care and Use Committee (QU-IACUC) approval and Qatar University Institutional Biohazard Committee (QU-IBC), Doha.

## 2. Materials and Methods

### 2.1. Smoking Machine Protocol and Water Pipe Preparation

**Figure 1:**
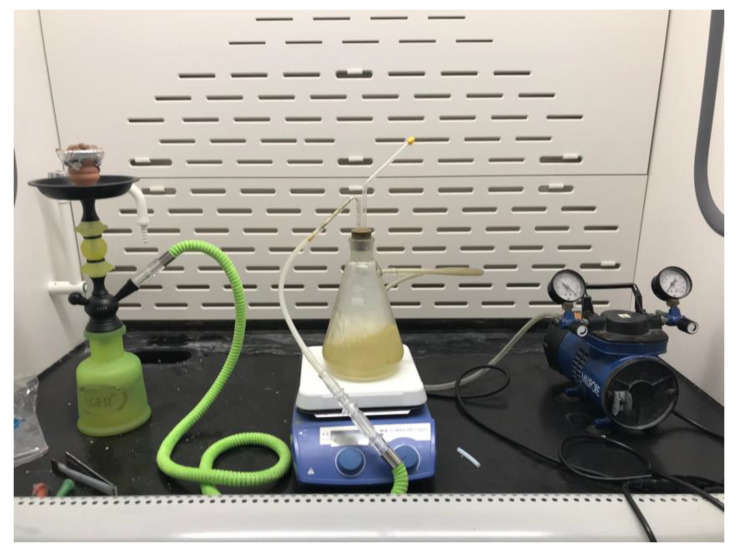
A new waterpipe smoking (WPS) setup used to collect smoke residue.

The standard Aleppo smoking protocol was modified using a new set up [21,22], as shown in Figure 1. Briefly, the WP apparatus was connected to a pump via an Erlenmeyer flask containing ethyl acetate (EtOAc). Ten grams of “Two Apples” brand tobacco was padded in the head and covered with perforated aluminium foil to allow air passage. A charcoal, “Three Kings” brand quick-light briquette, was ignited and placed on top of the head at the beginning of the smoking session. Water in the water bowl was changed at the beginning of every smoking session. The smoke collected from each run was passed by a slight negative pressure through a glass tube and collected in EtOAc. The collected EtOAc was evaporated under reduced pressure. Obtained residue was flushed with nitrogen gas until fixed weight, then stored in amber-colored vials and kept in the fridge until used. Before each experiment, collected WPS residue was dissolved in DMSO at concentrations of 1 mg/mL.

### 2.2. Zebrafish Maintenance and Microscopy

Wild-type AB zebrafish was kept at 28 °C temperature with a constant photoperiod of 14-h light, 10-h dark, with food (brine shrimp) supplied twice a day (Biomarine, Aquafauna Bio-Marine, Hawthorne, CA, USA). Zebrafish eggs were collected, and dead embryos were removed an hour after collection. The embryos were divided into six duplicate groups: control (1-phenyl 2-thiourea (PTU), negative control (0.1% DMOS), and the four experimental groups (50, 100, 150, 200 µg WPS extract dissolved in DMSO). The chosen concentrations were based on our previous in vitro work where two breast cancer cell lines, MCF7 and BT20, were used to explore the outcome of WPS [23]. In this study, the test was initiated soon after fertilization of the eggs and terminated 96 h after exposure. The embryos were immersed in test solutions before cleavage of the blastodisc stage, by the 16 cell-stage (2 hpf). All groups were examined at specific times 24, 48, 72 and 96 hpf depending on the development timeline of zebrafish, using specific equipment depending on the experiment running. Only Wild-type AB zebrafish embryos less than five days old were used for all experiments.

### 2.3. Developmental Toxicity

Treated and untreated embryos were examined 24 h post-fertilization. We used a Zeiss stereo discovery V8 Microscope equipped with Hamamatsu Orca Flash high-speed camera and a workstation equipped with HCImage software for opacity developmental endpoints. After the checkpoint (24, 48, 72 hpf), necrotic embryos were removed to protect the living embryos from contamination. Developmental abnormalities were photographed in exposed embryos along with their control. The survival rate was determined by counting the number of dead zebrafish embryos per group at 24 h post-fertilization (hpf) divided by the total number of treated embryos × 100. Danio Scope software measured the tail flicking assay (burst/min) to assess the potential neuro-muscular defect at 24-hpf. After that, the hatching percentage was calculated by counting the number of hatched embryos per group divided by the total number of treated embryos X 100. This was repeated at 72 hpf.

### 2.4. Behavioural and Locomotion Assays

Three days post fertilization; embryos were separated individually in E3M in a 96-well flat-bottomed plate then left in an incubator for an hour to acclimatize. After acclimatization, the plate was put in the Viewpoint Zebralab system (Noldus Information Technology, Wageningen, The Netherlands) set at 28 °C and illuminated with white light for an adaptation period of 10 min dark, 10 min light, for 50 min. The EthoVision XT 11.5 software was programmed according to the protocol needed to record individual locomotor activity. Arena and detection settings were adjusted to achieve optimal tracking. After the experiment was completed, the larvae were ethically euthanized. Data were exported to Excel and plotted using GraphPad Prism V8.

### 2.5. Cardiac Toxicity: Assessment of Cardiac Function

#### Live Imaging of Zebrafish

Zebrafish heart is composed of two chambers, one ventricle and one atrium. They have two major blood vessels: the dorsal aorta (DA) and the posterior cardinal vein (PCV) [24,25]. We measured several heart function/hemodynamics parameters, including cardiac output (CO), blood flow, blood velocity, vessel diameter, and heartbeat, by tracking red blood cells (RBC) movements in the DA and PCV from the trunk of the embryos. To do so, we used Danioscope (Noldus Information Technology Inc., Wageningen, The Netherlands), and we utilized MicroZebraLab blood flow from Viewpoint (version 3.4.4, Lyon, France) [26,27].

At 72 hpf, we used 3% methylcellulose to fix embryos from each treated and control group. We visualized them using Zeiss SteREO Discovery V8 Microscope equipped with Hamamatsu Orca Flash high-speed camera and a workstation equipped with HCImage software. A 10-s bright field video of the beating heart and tail was recorded for each embryo at 100 frames per second (fps) and 100× magnification.

To estimate the frictional sheer stress levels in the cardiovascular system, we used blood velocity measurements. Sheer stress (τ, dynes/cm^2^) was calculated using this formula τ = (4 μV_mean)/D, where µ is the blood viscosity (dynes/cm^2^), V is the average blood velocity (µm/s), and D is the vessel diameter (µm). Cardiac output (CO, mL/min, also known as flow rate), was measured using this formula F = V·A, where V is the average blood velocity (µm/s), and D is the vessel diameter (µm).

### 2.6. Cardiac Failure Markers Expression

RT-qPCR was performed to check the expression of two critical heart failure markers; atrial natriuretic peptide (ANP/NPPA), brain natriuretic peptide (BNP/NPPB) [28,29,30]. First, RNA was extracted from zebrafish embryos, both treated and control groups (20~30 each), using IBI DNA/RNA/Protein Extraction Kit (IBI Scientific -r IB47702, Dubuque, IA, USA) according to the manufacturer’s instructions. Then, we used SuperScript™ IV VILO™ Master Mix kit (Thermo Fisher Scientific 11756050, Waltham, MA, USA) according to the manufacturer’s instructions. Briefly, 1 μg of RNA for all samples was used to synthesize the first-strand cDNA. This was followed by a Quantitative analysis of specific mRNA expression using TaqMan^®^ Fast Advanced Master Mix (Applied Biosystems^®^, Waltham, MA, USA). Specific primers and probes designed (Applied Biosystems^®^, Waltham, MA, USA) to detect genes of interest: ANP/NPPA and BNP/NPPB (APGZVJD Catalog#: 4331348, APGZEPV Catalog#: 4331348) were used. The signal was read using RT-qPCR (QuantStudio™ 6 Flex RT-qPCR System). The relative quantity was calculated based on the 2-ΔCт method (Rao et al., 2013), and the fold change was measured in reference to the geomean of a group of housekeeping genes *B2M*.

### 2.7. Detection of Apoptotic Cells Using Acridine Orange (AO)

In this examination, we use acridine orange (AO) staining joined with Gen5™ Microplate reader and Imager programed to measure apoptotic cells in zebrafish embryos. Embryos were placed in 10 µg/mL of AO (Sigma, St. Louis, MO, USA) in E3 media. After 60 min of staining, embryos were washed three times in E3 media. After staining, embryos were transferred to round-bottom 96-well plates for imaging (Corning, catalogue #4520, Corning, NY, USA). Fluorescence was measured at excitation wavelength 490 nm and emission wavelength 520 nm. Embryos without acridine orange staining were used to determine baseline fluorescence. The fluorescence value was expressed as relative fluorescence units (RFU 5 fluorescence reading of experimental group minus baseline reading of control group).

### 2.8. Statistical Analysis

Statistical analysis was performed using GraphPad Prism 8 software. Distribution was determined using Kolmogorov–Smirnov normality test. Parametric data were analyzed using one way-ANOVA with Sidak post-hoc test, two-way-ANOVA with Dunnett test and unpaired two-tailed *t* test. While nonparametric data were analyzed using the Kruskal-Wallis test with Dunn’s post-hoc test (20 embryos were used in each group; the experiment was performed in triplicate). A *p*-value statistical significance was marked as follows * *p* < 0.05, ** *p* < 0.01, *** *p* < 0.001 and **** *p* < 0.0001.

## 3. Results

### 3.1. Developmental Toxicity

#### 3.1.1. Survival Assay (24 hpf)

**Figure 2:**
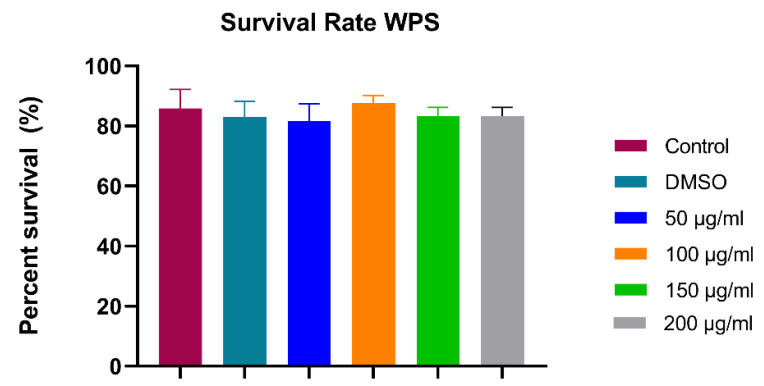
The survival rate of control embryos and experimental group at 24 h post-fertilization. All data are presented as mean ± SEM (20 embryos were used in each group; the experiment was performed in triplicate). One-way ANOVA with Sidak post hoc test was used to compare the differences between groups.

The survival rate at 24 hpf in control and DMSO groups was about 85.8% and 83%, respectively. There were no significant differences in mean survival rate between the four WPS concentrations (50, 100, 150 and 200 µg/mL) and the control group (Figure 2). No malformations were observed across all groups.

#### 3.1.2. Hatching Rate (48 hpf)

**Figure 3:**
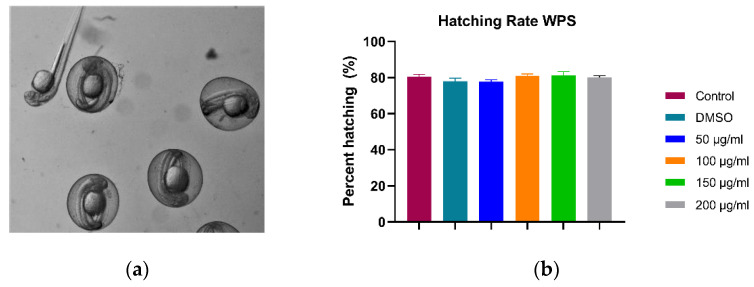
The effect of WPS on the hatching of zebrafish embryos at 48 hpf. (**a**) Representative image of embryos displaying hatched and unhatched embryos at 48 hpf. (**b**) The effect of WPS on the hatching percentage of the experimental groups at 48 hpf. All data are presented as mean ± SEM (20 embryos were used in each group; the experiment was performed in triplicate). One-way ANOVA with Sidak post hoc test was used to compare the differences between groups.

The hatching rate is the number of hatched embryos from their chorion, divided by the total number of survived embryos at 48 h post-fertilization. Our data showed that WPS treatment does not affect the hatching rate. As shown in Figure 3, there was no significant difference between the controls and the four WPS concentrations used in our study. The hatching rate was averaged at ~80% for all groups, which indicates that WPS has no potential effect on the neuromuscular system.

#### 3.1.3. Tail Flicking Assay (24 hpf)

**Figure 4:**
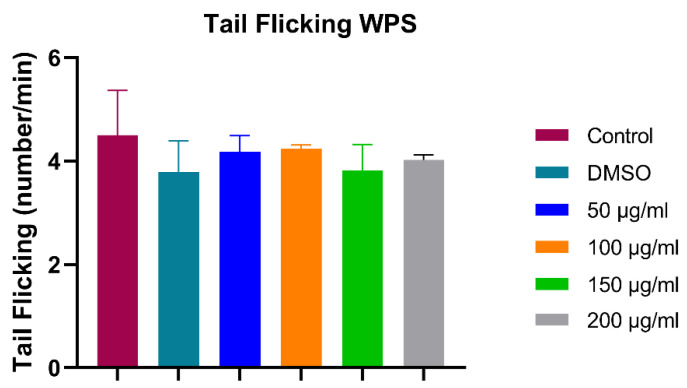
Assessment of potential neuromuscular toxicity at 24-hpf by tail flicking assay. All data are presented as mean ± SEM (50 embryos were used in each group; the experiment was performed in triplicate). One-way ANOVA with Sidak post hoc test was used to compare the differences between groups.

Tail flicking is the number of tail flicking of the embryos inside their chorions at 24 h post-fertilization counted for ten seconds and multiplied by 6 to complete a minute. As shown in Figure 4, The difference between various groups does not follow a specific trend. The range between the highest and lowest WPS treated groups was insignificant compared to control, confirming that WPS has no potential effect on the neuromuscular system.

### 3.2. Behavioural and Locomotion Assays (96 hpf)

We performed a locomotion assay to support the previous embryo tail flicking under the effect of WPS on the neuromuscular system. Locomotion assay was performed on 96-h post-fertilization for all groups; by measuring each embryo’s average and total distance in mm when exposed to alternating light/dark episodes.

**Figure 5:**
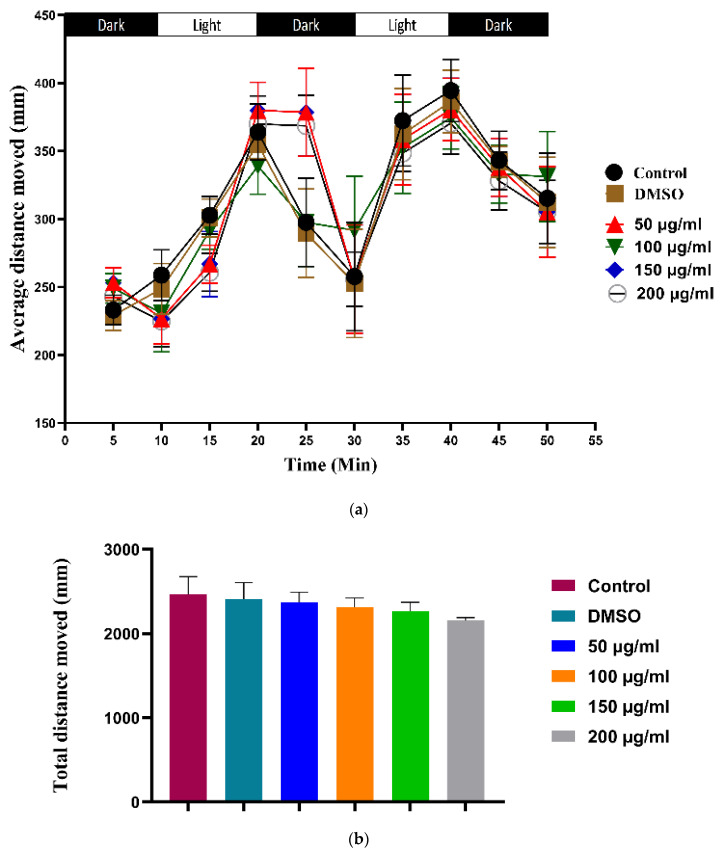
Behavioral and locomotion assays of the experimental group at 96 h post-fertilization. (**a**) Average distance moved by embryos; (**b**) Total distance moved by embryos. Data of the locomotion test are represented as mean ± SEM. (20 embryos were used in each group; the experiment was performed in triplicate). The analysis was done by a two-way-ANOVA test.

As shown in Figure 5, there is no significant difference between the distance moved by treated embryos from any of the four WPS concentrations and the control group. Moreover, the results show a regular motion pattern for treated zebrafish groups that mimic those of the control; their movement increased in light and decreased in the dark.

### 3.3. Cardiac Toxicity: Assessment of Cardiac Function

#### Live Imaging of Zebrafish (72 hpf)

**Figure 6:**
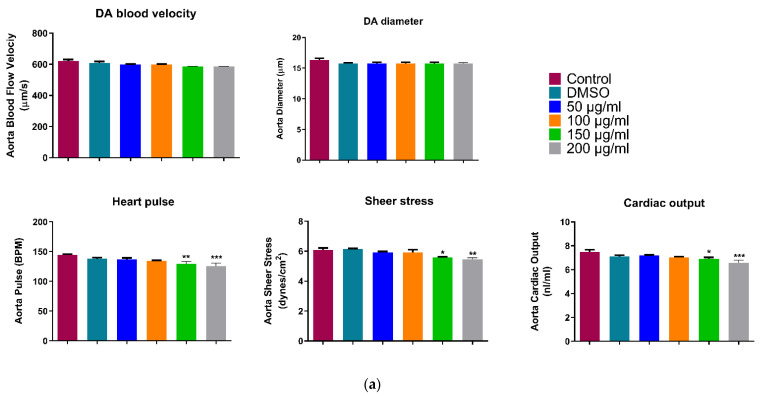

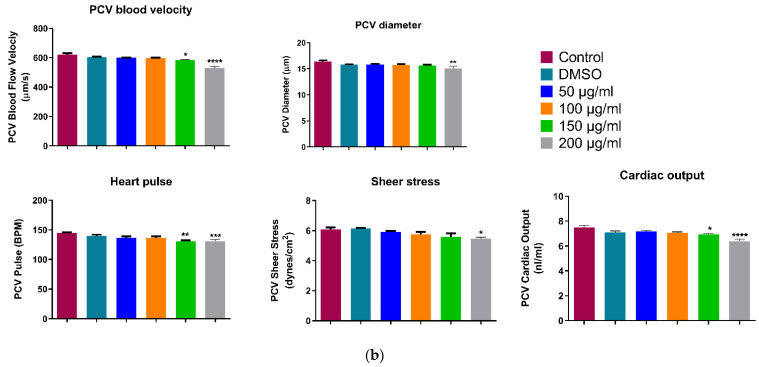
Assessment of the cardiac function of the experimental groups at 72 h post-fertilization (**a**) DA and (**b**) PCV blood flow analysis. All data are presented as mean ± SEM (6 embryos were used in each group; the experiment was performed in triplicate). The analysis was done by one-way ANOVA with Sidak post hoc test. * *p* < 0.05, ** *p* < 0.01, *** *p* < 0.001 and **** *p* < 0.0001.

We measured the cardiac function parameters on the two main blood vessels of Zebrafish dorsal aorta (DA) and Posterior Cardinal Vein (PCV). Cardiac output and blood flow analyses for both main blood vessels showed approximately the same trend. As shown in Figure 6a a slight rhythmic decrease can be seen with the increase in WPS concentration in all five measured parameters. However, there was no significant difference in blood velocity, DA diameter, heart pulse, sheer stress and cardiac output between the controls and 50, 100 µg/mL WPS concentrations (Figure 6a). The decrease in DA diameter, as well as blood velocity, was significantly prominent at the highest two concentrations of WPS treated groups (150 & 200 µg/mL), which in turn significantly reduced the overall cardiac output by 0.8-folds (*p* < 10^−3^) and 0.9 folds (*p* < 0.05), respectively. On the other hand, The 200 µg/mL WPS concentration group showed a significant decrease in all five parameters in the PCV blood analysis (Figure 6b). Collectively, our data suggested that WPS might have a harmful effect on zebrafish cardiac development.

### 3.4. Cardiac Failure Markers Expression (72 hpf)

**Figure 7:**
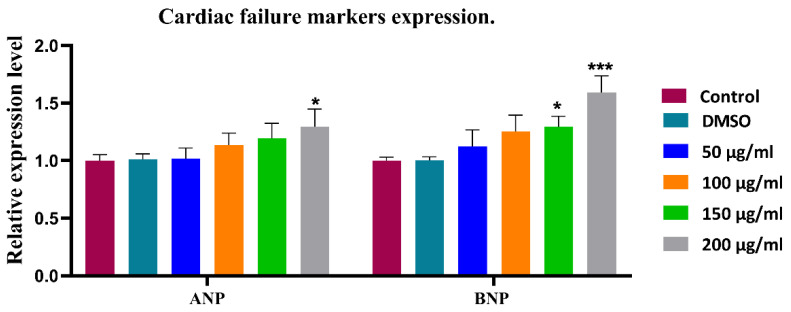
Cardiac failure markers expression of experimental groups at 72 h post-fertilization. All data are presented as mean ± SEM (experiments were carried in triplicate). The analysis was done by two-way ANOVA with Sidak post hoc test. * *p* < 0.05 and *** *p* < 0.001.

To further explore the effect of WPS on the cardiac functions at the molecular level, we investigated the expression of cardiac failure markers *ANP/NPPA* and *BNP/NPPB* in all experimental groups at 72 h post-fertilization. Our findings suggest that WPS exposure affect both cardiac failure markers expression. There was a steady increase in the expression of ANA and BNP with the increase in WPS concentration (Figure 7). WPS highest concentration (200 µg/mL) was associated with a significant increase in the expression of *ANP/NPPA* by 1.3-fold (*p* < 0.05) and *BNP/NPPB* by 1.6 fold (*p* < 10^−3^).

### 3.5. Acridine Orange Stainig for Apoptosis (72 hpf)

**Figure 8:**
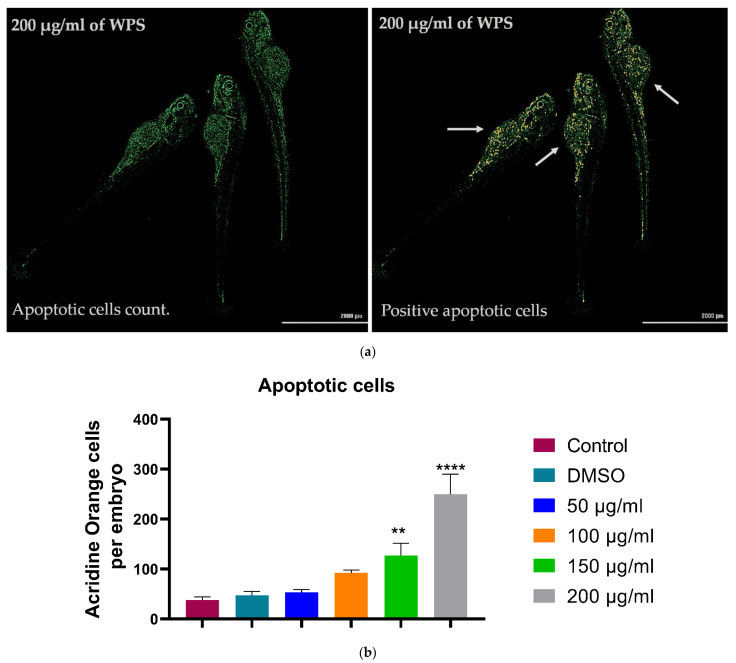
Quantitative apoptosis for apoptotic cells using Acridine Orange (AO). (**a**) Repetitive image of embryos exposed to highest WPS concentration (200 µg/mL) stained with acridine orange and showing positive apoptotic cells. (**b**) Quantitative analysis using a microplate assay revealed that apoptosis increased in a dose-dependent manner. All data are presented as mean ± SEM (experiments were carried 3 times in triplicate). The analysis was done by one-way ANOVA with the Dunnett test. ** *p* < 0.01 and **** *p* < 0.0001.

In order to investigate if WPS treatment would affect apoptosis in zebrafish embryos, staining with acridine orange was used. When bound to dsDNA, the dye emits green fluorescence and strongly binds to fragmented DNA, resulting from the apoptosis machinery. Thus, selective labelling of cells undergoing apoptosis is achieved. As shown in Figure 8, WPS treatment affects zebrafish embryos, as there is a steady increase in the number of apoptotic cells with increased concentrations of WPS in a dose-dependent manner. The dose-dependent effects of WPS were quantitated using a fluorescence microplate reader (Figure 8). Our results show that apoptosis significantly appeared at the highest concentrations (150 & 200 µg/mL) of WPS, which is associated with a significant increase in apoptosis of 2.7-folds (*p* < 10^−2^) and 5.3-folds (*p* < 10^−4^), respectively.

## 4. Discussion

Numerous studies found a link between tobacco smoking and severe diseases such as cancers, cardiovascular, respiratory diseases, and stroke [1,31,32]. However, the effect of tobacco and, more specifically, WPS on the embryos is not yet fully understood. In this study, we explored for the first time the effect of WPS on the early stages of Zebrafish embryo, a low-cost, high-throughput in vivo model. In this study we exposed zebrafish embryos to different concentrations of WPS solution and measured acute, developmental, cardiac, and behavioral toxicity (neurotoxicity).

Our results showed no significant effect of WPS on the embryonic development of zebrafish except for the expression of cardiac failure markers. This indicates that the effect of WPS is chronic and requires continuous use to manifest its effect on health. Furthermore, WPS had no acute lethal effect on zebrafish embryos as no change in survival and hatching rates was observed between the control and exposed groups. There was also no significant difference in tail flicking, which illustrates that the neuromuscular system was not affected by WPS after three days of exposure.

To further support the tail flicking finding, we performed a locomotion assay, where WPS treated embryos were exposed to alternating light/dark episodes. In most cases, WPS exposed zebrafish embryos exhibited normal movement when they were stimulated by light. WPS had no effect on the locomotion assay as the behaviour of WPS treated zebrafish groups mimicked the behaviour of those in the control group; their movement increased in the light periods and decreased in the dark. Collectively, these findings showed that WPS did not affect the neuromuscular system of the zebrafish afterthree days of exposure. However, we cannot rule out the possible effect of more prolonged chronic exposure or higher concentrations of WPS on the neuromuscular system as they were not addressed in this study, but warrant further investigation.

Moreover, to assess the effect of WPS on the cardiovascular system of zebrafish embryos, five measurements were taken on the two main blood vessels of the zebrafish. A slight rhythmic decrease was demonstrated for the dorsal aorta with a progressive increase in WPS concentrations. This indicates that a high concentration of WPS can affect blood velocity even with a short exposure time. Our results show that exposure to WPS had no considerable acute or short-term effect. However, this slight change in blood vessels measurement is predictive of future chronic effects. These results are in concordance with earlier reports by Palpant et al. (2015), who used the zebrafish to examine the cardiac effects of developmental exposure to purified nicotine, conventional cigarette smoke or e-cigarette vapour during the first three days of development [20]. Overall, they showed that tobacco and e-cigarette smoke-exposed embryos had significantly more heart defects compared to the purified nicotine group. The heart defects in embryos exposed to tobacco and e-cigarette smoke were also more severe than in embryos exposed to nicotine alone and exhibit a broader spectrum of cardiac developmental defects [20]. Both types of cigarettes decrease expression of cardiac transcription factors in cardiac progenitor cells, suggesting a persistent delay in differentiation [20].

For the prediction of a heart failure before it happens, some cardiac markers can be measured includingANP/NPPA and BNP/NPPB [28,29,30]. Natriuretic peptides are quantitative markers of cardiac hemodynamic stress and heart failure. Therefore, these peptides serve as good markers to assess patients with suspected acute heart failure and help with the diagnosis. ANP stimulates the kidney to increase its excretion and cause blood vessels relaxation [33,34]. When the blood volume increases, BNP or Ventricular Natriuretic Peptide is secreted in heart ventricles by cardiomyocytes, causing stretching to the ventricles [35,36].

Here, the gene expression for these two markers was measured quantitatively to assess the load on the heart. There was an increase in cardiac failure markers with increased exposure to WPS. Surprisingly, even within a short time of exposure, the highest WPS concentration (200 µg/mL) had a significant effect on the expression of *ANP/NPPA* by 1.3-fold (*p* < 0.05) and *BNP/NPPB* by 1.6-fold (*p* < 10^−3^). The increase in *BNP/NPPB* levels at 72 hpf might slow down the progress of morphological and functional changes in the zebrafish heart, such as reduced heart rate, shape and blood velocity. This indicates potential heart disorder due to WPS exposure in the absence of specific phenotype changes at this stage. To assess the cardiac status generally, we can speculate that the decrease in cardiac output, blood velocity, heart pulse measurements, and the steady increase in cardiac failure markers in the 200 ug/mL group show the extent of WPS affects the cardiovascular system. Even though we did not pinpoint specific WPS chemical component(s) with regards to these observed effects on the heart, the aim herein is to prove the toxic effect of WPS on embryonic development, which has been demonstrated.

These findings are aligned with a previously published study which reported that *ANP/NPPA* and *BNP/NPPB* were absent in healthy adult hearts and were upregulated under hypertrophic stimuli [37]. Other studies showed that *ANP/NPPA* knocked out mice had hypertrophy and hypertension under both resting and pressured conditions as compared to wild-type control mice [38,39]. This suggests that natriuretic peptides (*ANP/NPPA* and *BNP/NPPB)* may slow down the progress of morphological and functional changes in the developing heart but may not prevent it.

Comparing the significant cardiac toxicity of WPS with its insignificant neuromuscular effect, we concluded that the cardiovascular system in the zebrafish embryos has a faster response to toxins. Collectively, this study provides evidence that WPS might have potentially serious adverse effects on the cardiovascular system at the developmental stages of zebrafish embryos.

Apoptosis, or programmed cell death, is an important part of development and pathology invertebrates and is involved in normal cell turnover, eliminating unnecessary cells after differentiation, and being activated in response to environmental stress. Moreover, the process of apoptosis during development is very well conserved between zebrafish and higher animals, making the zebrafish a suitable model to study embryotoxic effects of environmental stimuli [40,41].

In this study, we investigated the effect of WPS by conducting an automatic assessment of the relative fluorescence of larger pools of embryos using digital image analysis. The dose-dependent effects of WPS showed that apoptosis significantly appeared at the highest concentrations, which indicates a harmful effect of WPS at the cellular level of developing embryos. Stress-induced apoptosis is thought to be a factor in the pathophysiology of malformations during embryogenesis [40].

Zebrafish model extends the data generated from the in vitro and cell culture models currently employed for WPS toxicity testing and could be useful in probing the changes in organismal toxicity relating to WPS design which is not apparent in cell culture assays.

## 5. Conclusions

Water pipe smoking has been a progressive health problem for a decade. Its consumption increases, especially among women, thus potentially affecting the embryonic development in pregnant women who smoke or are exposed to secondhand smoking. In this regard, our group recently reported the very toxic effect of WPS using the chicken embryo model [10]. At the same time, zebrafish presents another excellent model to study WPS toxicity owing to its several advantages. Acute exposure of zebrafish embryos to WPS did not affect the neuromuscular system but highly disturbed known cardiac parameters. Nevertheless, future studies using different animal models are warranted to pinpoint the effect of WPS and its mechanism on normal fetal development.
